# Vitamin D modulates biliary fibrosis in ABCB4-deficient mice

**DOI:** 10.1007/s12072-014-9548-2

**Published:** 2014-06-21

**Authors:** Katrin Hochrath, Caroline S. Stokes, Jürgen Geisel, Marion J. Pollheimer, Peter Fickert, Steven Dooley, Frank Lammert

**Affiliations:** 1 Department of Medicine II, Saarland University Medical Center, Kirrberger Str. 100, 66421 Homburg, Germany; 2 Institute of Clinical Chemistry and Laboratory Medicine, Saarland University Medical Center, Homburg, Germany; 3 Insititute of Pathology, Medical University Graz, Graz, Austria; 4 Division of Gastroenterology and Hepatology, Department of Internal Medicine, Medical University of Graz, Graz, Austria; 5 Division of Molecular Hepatology-Alcohol Associated Diseases, Department of Medicine II, Medical Faculty Mannheim, University of Heidelberg, Mannheim, Germany

**Keywords:** ABC transporters, Cholecalciferol, Chronic cholangitis, Fibrogenesis, Liver injury

## Abstract

**Purpose:**

Impaired vitamin D receptor signaling represents an aggravating factor during liver injury, and recent studies suggest that vitamin D might exert a protective role in chronic hepatobiliary diseases. We hypothesized that vitamin D supplementation would ameliorate liver fibrosis in ATP-binding cassette transporter B4 knockout (*Abcb4* ^−*/*−^) mice as a preclinical model of sclerosing cholangitis.

**Methods:**

*Abcb4* ^−*/*−^ and wild-type mice were fed a regular chow diet (600 IU vitamin D/kg food) or diets with lower (100 IU/kg) and higher (2,400 IU/kg) vitamin D concentrations for 12 weeks. Serum 25-hydroxyvitamin D concentrations were measured by chemiluminescence immunoassays. Liver injury and biliary fibrosis were assessed by liver enzyme activities, histopathology and hepatic collagen contents. Hepatic mRNA expression of markers for fibrosis, vitamin D and bile acid metabolism were analyzed by quantitative PCR.

**Results:**

Different vitamin D concentrations were observed depending on genotype and diet group, with *Abcb4* ^−*/*−^ mice on the control diet showing lower vitamin D concentrations compared to wild-type mice. *Abcb4* ^−*/*−^ animals on the low vitamin D diet demonstrated the most advanced liver fibrosis and highest hepatic collagen contents. Feeding *Abcb4* ^−*/*−^ mice a high vitamin D diet enriched serum vitamin D levels, lowered liver enzyme activities, altered expression levels of profibrogenic genes and ameliorated, in part, liver injury.

**Conclusions:**

This is the first report to demonstrate that fibrogenesis in the established *Abcb4* ^−*/*−^ model is influenced by vitamin D supplementation. Since vitamin D modulates sclerosing cholangitis in vivo, we speculate that sufficient vitamin D intake might improve liver damage and induce antifibrotic effects in chronic cholestasis in humans.

**Electronic supplementary material:**

The online version of this article (doi:10.1007/s12072-014-9548-2) contains supplementary material, which is available to authorized users.

## Introduction

Vitamin D deficiency occurs in up to 90 % of patients with chronic liver diseases, irrespective of the underlying etiology of liver injury [1, 2]. Moreover, in these patients, low vitamin D levels are associated with increased grades of necroinflammation and stages of fibrosis and a heightened mortality risk [3–6]. Despite these observations, and in particular the inverse correlation between vitamin D levels and the severity of liver diseases, to date few studies have investigated the beneficial and adverse effects of vitamin D supplementation in chronic liver diseases [7–9]. Therefore, data regarding the efficacy of vitamin D supplementation are still lacking, and preclinical models for assessing the effects of vitamin D are needed.

Vitamin D is a steroid hormone with pleiotropic effects that undergoes hydroxylation in liver and kidney [6]. The major circulating form, 25-hydroxyvitamin D, is bound to vitamin D-binding protein, whereas effects of 1α,25-dihydroxyvitamin D on target genes in many organs are mediated by a ligand-activated nuclear receptor, the vitamin D receptor (VDR) [10], which forms a heterodimer with the retinoid X receptor (RXR) to modulate processes and networks ranging from immune responses to mineral homoeostasis (Supplementary Fig. 1). In the liver, VDR expression is restricted to non-parenchymal cells and biliary epithelial cells [11]. Besides activation of VDR by vitamin D, lithocholic acid and its derivatives have been demonstrated to function as VDR ligands and activators [12]. Furthermore, regulation of bile acid synthesis and enzymes responsible for bile acid detoxification are influenced by vitamin D-VDR-signaling [13–15]. These findings collectively point to a potential modulatory role of vitamin D-VDR signaling in biliary-type liver injury.

Hence, we hypothesized that vitamin D supplementation ameliorates liver fibrosis in vivo by phenotyping *Abcb4* (ATP-binding cassette transporter, subfamily B, member 4) knockout (*Abcb4* ^−*/*−^, also known as *Mdr2* ^−*/*−^) mice, an established and highly reproducible model of sclerosing cholangitis [16–18]. *Abcb4* ^−*/*−^ mice lack the hepatocanalicular phosphatidylcholine floppase ABCB4 and develop sclerosing cholangitis and liver fibrosis [19]. The disease caused by mutations of the orthologous human gene is called progressive familial intrahepatic cholestasis (PFIC type 3), which resembles primary sclerosing cholangitis.

## Materials and methods

### Animals

FVB/N-*Abcb4* ^*tm1bor*^ mice and FVB/NJ control mice were obtained from the Jackson Laboratory (Bar Harbor, ME, USA). The mice were housed and bred in individually ventilated cages with a 12-h light-dark cycle under incandescent lighting free from UVB radiation. Temperature and humidity were regulated at 22 ± 1 °C and 55 ± 5 %, respectively. Water and food were provided ad libitum. Mice were genotyped by polymerase chain reaction (PCR) of tail DNA using *neo* (5′-CTT GGG TGG AGA GGC TAT TC-3′; 5′-AGG TGA GAT GAC AGG AGA TC-3′) and *Abcb4* (5′-CAC TTG GAC CTG AGG CTG TG; TCA GGA CTC CGC TAT AAC GG-3) specific primer pairs. The PCR reaction included PCR buffer (Applied Biosystems, Darmstadt, Germany), 2 mM MgCl_2_, 10 μM dNTPs, 10 μM primer, 1.25 U *Taq* DNA polymerase (Invitrogen, Darmstadt, Germany) and 20–100 ng DNA in 25-µl reactions. PCR cycling conditions were 94 °C/30 s, 55 °C/60 s and 72 °C/30 s for 35 cycles and a final extension step of 10 min at 72 °C.

The animal experiments were performed with permission from the federal state of Saarland according to §8 of the German Law for the Protection of Animals and the Directive 2010/63/EU of the European Parliament. All institutional and national guidelines for the care and use of laboratory animals were followed.

### Diets

The vitamin D diets were commenced after weaning in the 4th week of age, coinciding with the initiation of liver pathology including sclerosing cholangitis, which at this point is not yet fully developed [20]. Wild-type controls and *Abcb4* ^−*/*−^ mice of both sexes were divided into three groups, with each receiving a different diet: The control group was fed a regular chow diet containing 600 IU vitamin D (cholecalciferol)/kg food, which is based on the established nutrient requirements for laboratory mice [21]. The low vitamin D group obtained a diet with 100 IU vitamin D/kg, and the high vitamin D diet group was fed a diet enriched with 2,400 IU vitamin D/kg. All diets were given for 12 weeks. With the exception of vitamin D content, all three diets were otherwise equal in nutrient composition and total energy, and were obtained from Altromin (Lage, Germany; see Supplementary Tables 1, 2 and 3 for detailed dietary composition). In total, 113 animals were analyzed, with a minimum of 15 mice per genotype and diet group. Table 1 provides the number of animals analyzed per genotype, sex and diet group. Food intake was controlled by monitoring weekly food consumption per cage and did not differ (3–4 g/day) among the groups. Survival rates were 100 %.

**Table 1:** Numbers of animals analyzed per genotype, sex and diet group

	FVB-*Abcb4* ^−*/*−^		FVB/NJ wild-type	
	Female	Male	Female	Male
Low vitamin D diet (100 IU/kg)	11	9	6	9
Control vitamin D diet (600 IU/kg)	10	7	10	9
High vitamin D diet (2,400 IU/kg)	12	11	9	11

### Phenotypic characterization of biliary fibrosis

For histopathological evaluation, liver samples were preserved in 4 % neutral-buffered formaldehyde solution at 4 °C and embedded in paraffin. Paraffin sections (2 μm) were stained with haematoxylin-eosin (H&E) or Sirius red for the detection of collagen. The stages of liver fibrosis and relative collagen areas were assessed by a pathologist blinded to the study protocol using a semiautomatic system for image analysis (Stingray F146C IRF Medical camera, ½″ type progressive scan CCD, Germany) and HistoQuant image morphometry software (3DHistech, Budapest, Hungary). Hepatic fibrosis was staged (F-score) using a scale adapted from the Batts and Ludwig as well as Ishak scoring systems [22, 23]. The F-scores are subdivided into five classes: 0 = no fibrosis; 1 = scatter periportal and perineoductular fibrosis; 2 = periportal, perineoductular fibrosis; 3 = periportal, perineoductular fibrosis with portal–portal septa; 4 = complete cirrhosis.

Relative collagen content was determined as the mean percentage of the collagen-stained area to the whole area (field of view). Therefore, we screened representative microscopic fields (magnification, 100×), which were randomly chosen from each liver section (avoiding arteries of >100 μm diameter) after setting a threshold capturing Sirius red-stained areas of collagen. In addition, collagen in liver was quantified by colorimetric measurement of the collagen-specific amino acid hydroxyproline, as described previously [24, 25].

### Serum biochemical assays

Blood was collected from the inferior vena cava after harvesting. Samples were left for 10 min at room temperature in darkness and centrifuged for 20 min at 2,000×*g*. Serum was stored at −80 °C until analysis. Serum calcium concentrations, alanine aminotransferase (ALT), aspartate aminotransferase (AST) and alkaline phosphatase (AP) activities were measured in the Cobas^®^ 8000 analyzer (Roche Diagnostics, Mannheim, Germany) by standardized methods following the recommendations of the International Federation of Clinical Chemistry. Serum 25-hydroxyvitamin D concentrations were determined using the chemiluminescence immunoassay LIAISON^®^ 25 OH Vitamin D TOTAL Assay (DiaSorin, Stillwater, MN, USA).

### Gene expression analyses by quantitative real-time PCR

Hepatic mRNA expression levels of individual genes were determined by quantitative real-time PCR (qPCR) (*TaqMan*, Applied Biosystems) using 1 μg RNA for reverse transcription and 18S RNA as endogenous control, with one cycle at 95 °C for 10 min, followed by 45 cycles at 95 °C/30 s and 60 °C/60 s. The sex-specific expression levels were calculated by the ΔΔct-method [26] in relation to counterpart wild-type controls (e.g., ΔΔct_*Col1α2*_ = Δct_*Col1α2*_ *Abcb4* ^−*/*−^ female on low vitamin D diet-mean Δct_*Col1α2*_ wild-type female on low vitamin D diet). The relative quotient (RQ, 2^−ΔΔct^) for each sample was normalized to sex-specific wild-type mice fed the control diet. The *TaqMan* (Applied Biosystems) expression assay IDs were *Col1α2*, Mm01165187_m1; *Cramp*, Mm00438285_m1; *Cyp2r1*, Mm01159413_m1; *Cyp7a1*, Mm00484150_m1; *Cyp8b1*, Mm00501637_s1; *Cyp27a1*, Mm00470430_m1; *Tgfb1*, Mm03024053_m1; *Timp1*, Mm00441818_m1; *Vdr*, Mm00437297_m1.

### Immunofluorescence microscopy for cytokeratin 19 and E-cadherin

Double immunofluorescence staining for cytokeratin 19 (CK19) and E-cadherin was performed on an acetone-fixed (−20 °C, 10 min) cryosection of liver tissue (4–6 animals per genotype and diet group). CK19 was detected using a monoclonal rabbit anti-Troma-III antibody (1:500) [27]. In addition, slides were incubated with a monoclonal rabbit antibody anti-E-cadherin (1:200; no. 3195, Cell Signaling Technology, Danvers, MA, USA). Secondary antibodies were conjugated to fluor 488-goat anti-rabbit (1:100) or tetramethylrhodamine isothiocyanate 565-anti-rabbit antibody (1:50) [19].

### Statistics

The results were analyzed using SPSS 20.0 (IBM, Ehningen, Germany). Quantitative data are presented as means ± standard errors (SE) or median and ranges, as appropriate. All data were analyzed for the effects of diet and genotype. Means were compared with Student's *t* tests or one-way analyses of variance (ANOVA), followed by post hoc Bonferroni correction. The medians of non-parametric data were compared with Mann–Whitney *U* or Kruskal–Wallis tests, respectively. Two-way ANOVA was applied to assess the interaction of diet and sex after exclusion of extreme values. Correlation coefficients were calculated according to Pearson or Spearman as appropriate. *p* values < 0.05 were considered significant.

## Results

### Vitamin D supplementation exerts no toxic effects in *Abcb4*^−/−^ mice

After weaning, all mice continued to develop normally and displayed no obvious signs of developmental or behavioral abnormalities. Body weight and liver-to-body weight ratios were not affected by dietary composition, with the exception of female *Abcb4* ^−*/*−^ mice on the high vitamin D diet displaying increased body weight compared to knockout mice on the control diet (24.6 ± 0.9 g vs. 20.2 ± 0.9 g, *p* < 0.01). Serum calcium levels were slightly higher in *Abcb4* ^−*/*−^ mice as compared to wild-type controls in all dietary groups, and no hypercalcemia was induced (Supplementary Fig. 2).

### Vitamin D serum concentrations depend on genotype, sex and diet

**Fig. 1:**
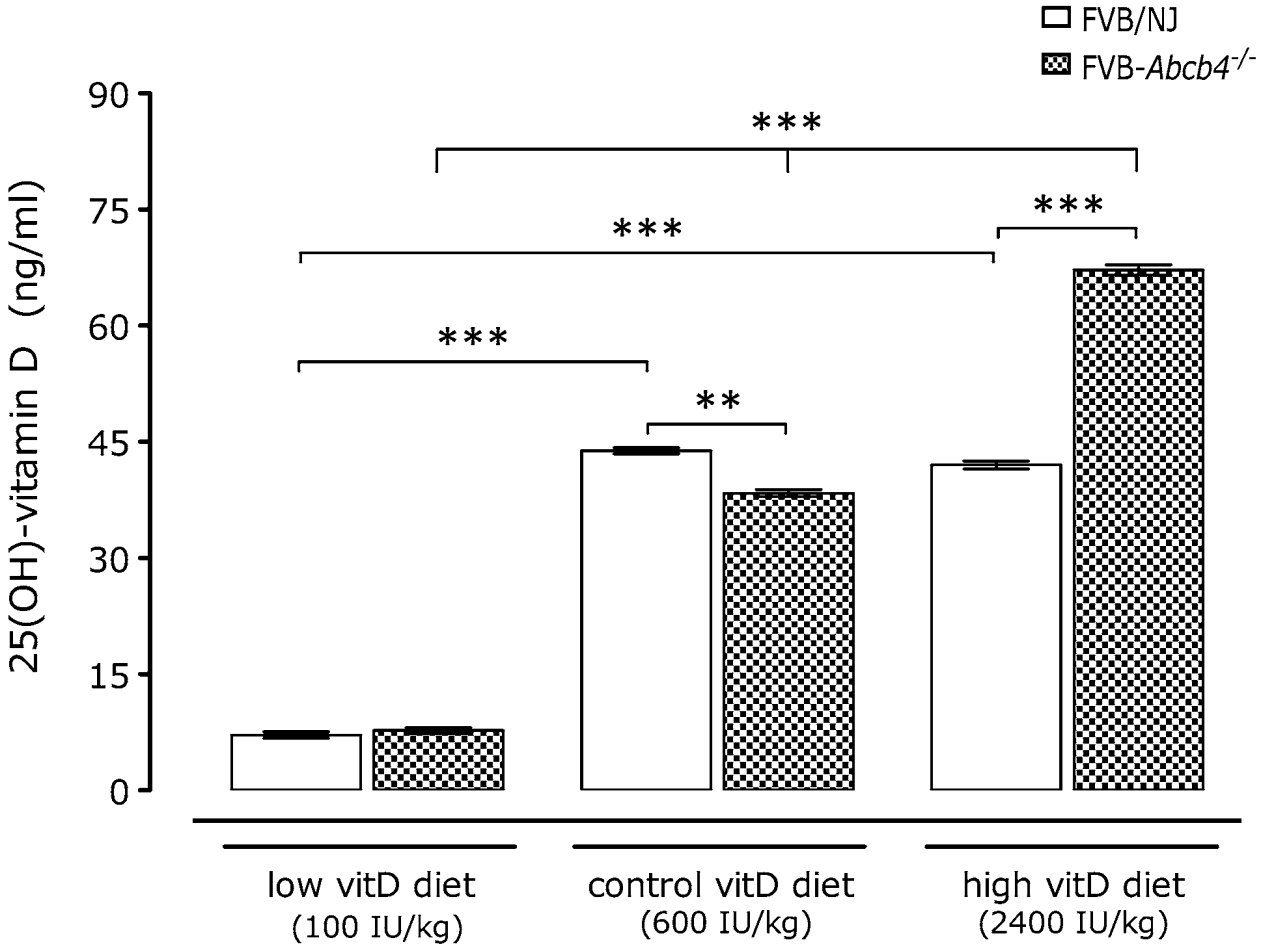
Mean Serum 25-hydroxyvitamin D concentrations in *Abcb4* ^−*/*−^ and wild-type mice fed different vitamin D diets for 12 weeks. **p < 0.01, ***p < 0.001

As shown in Fig. 1, mice displayed different serum vitamin D concentrations depending on genotype and diet group. On the low vitamin D diet, both strains displayed serum vitamin D levels <10 ng/ml. *Abcb4* ^−*/*−^ mice on the control diet showed significantly (*p* < 0.01) lower vitamin D concentrations compared to FVB/NJ wild-type mice on this diet (38.4 ± 1.9 vs. 46.8 ± 1.7 ng/ml). In contrast, *Abcb4* ^−*/*−^ mice on the high vitamin D diet demonstrated significantly (*p* < 0.001) increased vitamin D levels in comparison to wild-type counterparts (67.2 ± 2.4 vs. 42.0 ± 2.3 ng/ml). The vitamin D concentrations in female mice were significantly (*p* < 0.001) higher than in male mice in the high vitamin D group, and a significant (*p* < 0.01, two-way ANOVA) interaction between sex and diet was observed for vitamin D levels but not for other phenotypes.

### Vitamin D supplementation influences hepatic fibrosis in *Abcb4*^−/−^ mice

**Fig. 2:**
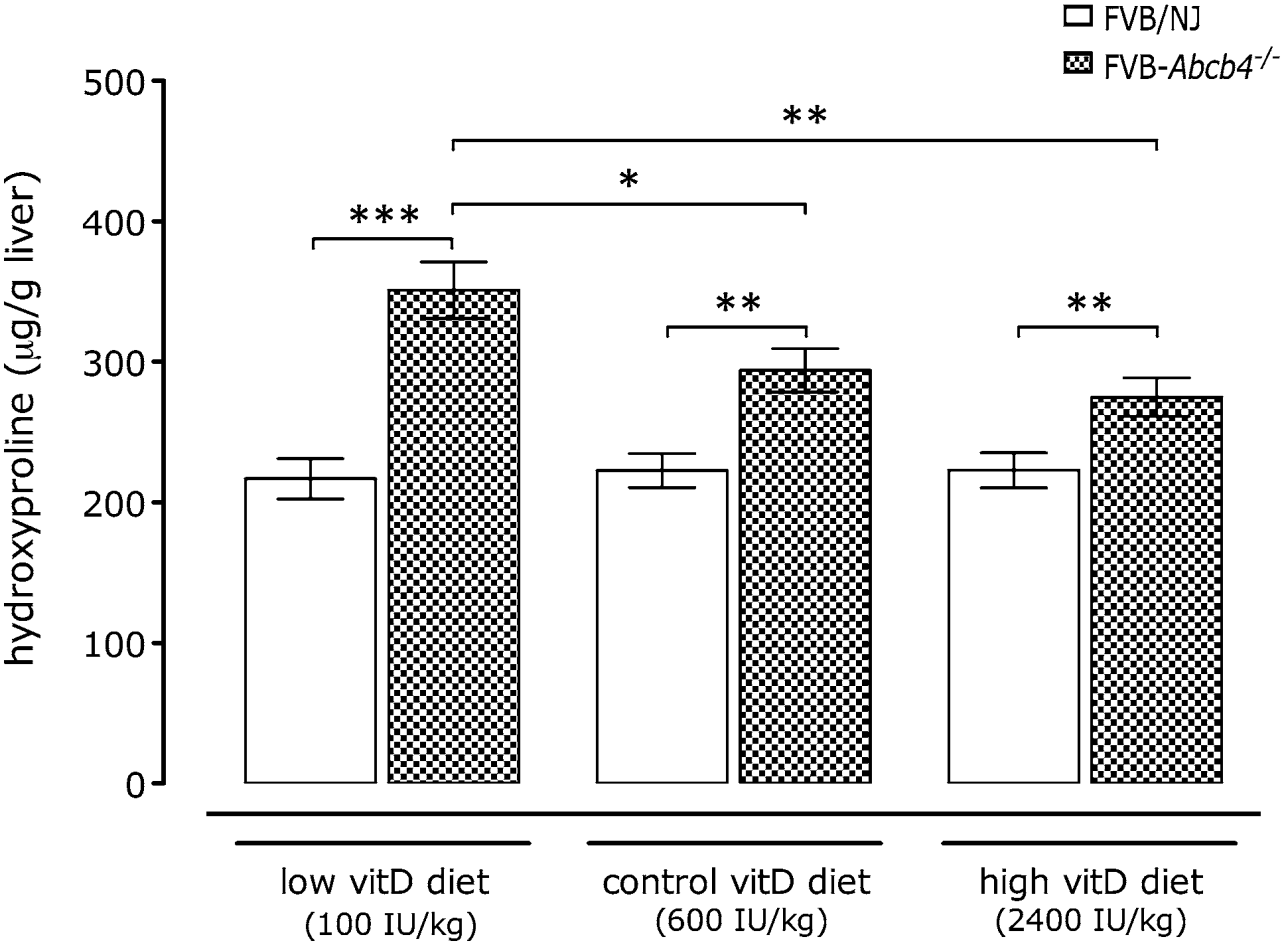
Mean hepatic collagen contents (±SE) as determined via the collagen-specific amino acid hydroxyproline in *Abcb4* ^−*/*−^ and wild-type mice fed different vitamin D diets for 12 weeks. *p < 0.05, **p < 0.01, ***p < 0.001

Figure 2 illustrates that the quantification of hepatic collagen contents revealed a significant (*p* < 0.01) increase in *Abcb4* ^−*/*−^ mice in all three diet groups as compared to controls. Hepatic collagen contents were highest in *Abcb4* ^−*/*−^ mice receiving the vitamin D-deficient diet and displayed a dose-related trend towards lower levels in knockout mice on the regular and the high vitamin D diets. In contrast, no impact of diet on hepatic collagen levels was detected in wild-type mice (Fig. 2).

Histopathological staging of liver fibrosis revealed no fibrosis (F0) in wild-type mice. Contrary, *Abcb4* ^−/−^ mice progressed to fibrosis stage F3. *Abcb4* ^−*/*−^ animals on the low vitamin D diet demonstrated significantly (*p* < 0.001) higher fibrosis scores (median F3, range F2–F3) compared with knockout mice fed the control or the high vitamin D diets (high vitamin D diet: median F2, range F1–F3; control diet: median F2, range F0–F3).

**Fig. 3:**
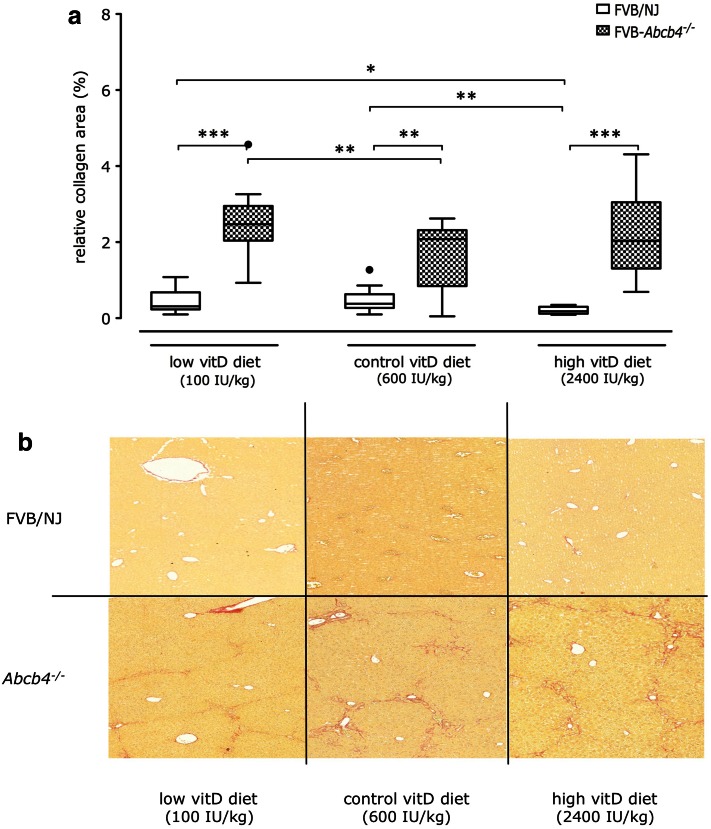
(**a**) Median relative collagen contents (with 1.5 interquartile ranges) as determined after Sirius red staining and (**b**) representative liver sections after Sirius red staining of livers from *Abcb4* ^−*/*−^ and wild-type mice fed different vitamin D diets for 12 weeks. *p < 0.05, **p < 0.01, ***p < 0.001. (Color figure online)

Panel A of Fig. 3 shows that these histopathological observations were reflected by the relative collagen areas, as determined after Sirius red staining. Representative liver sections stained with Sirius red are shown in Fig. 3b. As expected, we observed genotype-dependent differences, with increased collagen areas in *Abcb4* ^−*/*−^ mice as compared to wild-type mice. Collagen areas were significantly (*p* < 0.01) larger in *Abcb4* ^−*/*−^ mice receiving the low vitamin D diet in comparison to those on the control diet (2.47 %, range 0.93–4.57 vs. 2.08 %, range 0.05–2.62). The lowest collagen areas in *Abcb4* ^−*/*−^ mice were determined in animals fed the high vitamin D diet (2.03 %, range 0.69–4.31). Interestingly, wild-type mice on the high vitamin D diet also showed significantly (*p* < 0.01) reduced collagen areas (0.18 %, range 0.09–0.35) in comparison to mice on diets with lower vitamin D concentrations (control diet: 0.38 %, range 0.10–1.27; low vitamin D diet: 0.31 %, range 0.10–1.08).

**Fig. 4:**
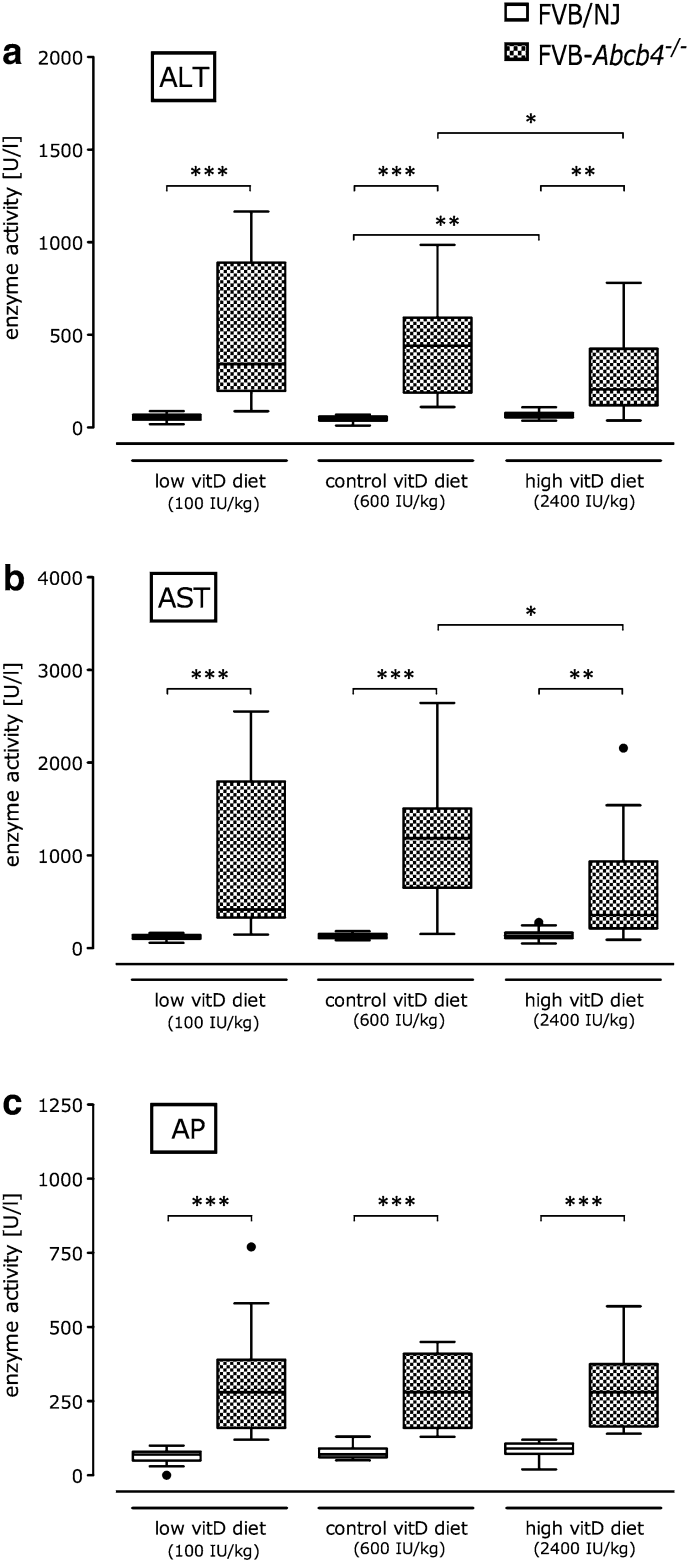
Serum liver enzyme activities. **a** Alanine aminotransferase (ALT), **b** aspartate aminotransferase (AST) and **c** alkaline phosphatase (AP) activities in *Abcb4* ^−*/*−^ and wild-type mice fed different vitamin D diets. *p < 0.05, **p < 0.01, ***p < 0.001

Serum parameters for liver damage and cholestasis (ALT, AST, AP) were significantly elevated in *Abcb4* ^−*/*−^ compared to corresponding wild-type mice in all diet groups (Fig. 4). Notably, enzymatic activities of both ALT and AST in *Abcb4* ^−*/*−^ mice significantly decreased on the high vitamin D diet as compared to knockout mice fed the control diet. Wild-type mice receiving the high vitamin D diet displayed elevated ALT activities in comparison to those on the control diet (68 U/l, range 37–314 vs. 48 U/l, range 11–77; *p* < 0.01). AP activities did not differ between diet groups in either knockout or wild-type mice (Fig. 4c).

### Dietary vitamin D affects hepatic expression of profibrogenic genes in *Abcb4*^−/−^ mice

**Fig. 5:**
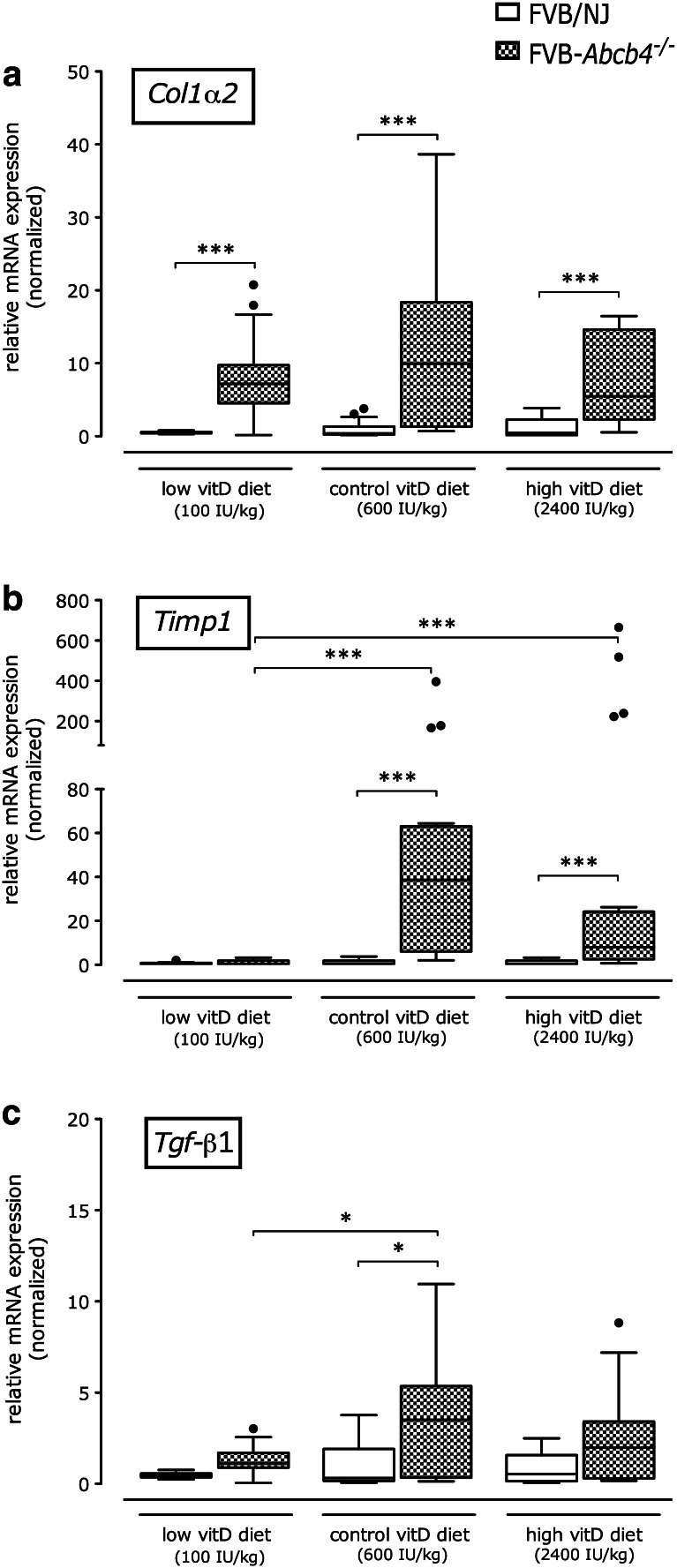
Relative hepatic mRNA expression levels of profibrogenic genes. **a** Collagen type I, α2-chain (*Col1a2*), **b** tissue inhibitor of matrix metalloproteinase 1 (*Timp1*) and **c** transforming growth factor-β1 (*Tgf-β1*) in *Abcb4* ^−*/*−^ and wild-type mice fed different vitamin D diets for 12 weeks. Data are normalized based on the expression of wild-type mice fed the vitamin D control diet (600 IU/kg food). *p < 0.05, **p < 0.01, ***p < 0.001

Panel A of Fig. 5 illustrates that hepatic steady-state mRNA expression levels of collagen type I (*Col1α2*) were significantly (*p* < 0.001) higher in ABCB4-deficient mice as compared to wild-type controls, irrespective of the diet. *Timp1* expression increased significantly (*p* < 0.001) in *Abcb4* ^−*/*−^ mice fed the control or the high vitamin D diets, but not in animals fed the low vitamin D diet as compared to corresponding wild-type mice (Fig. 5b). Panel C of Fig. 5 shows that a similar trend was observed for transforming growth factor (*Tgf*)-*β1* expression, with significantly (*p* < 0.05) reduced levels in *Abcb4* ^−*/*−^ mice on the low vitamin D diet as compared to the control diet. *Tgf*-*β1* expression correlated significantly (*ρ* = 0.92, *p* < 0.01) with expression of *Vdr* in *Abcb4* ^−*/*−^ mice.

### Diet and genotype modify the hepatic expression of *Vdr* and vitamin D hydroxylases

**Fig. 6:**
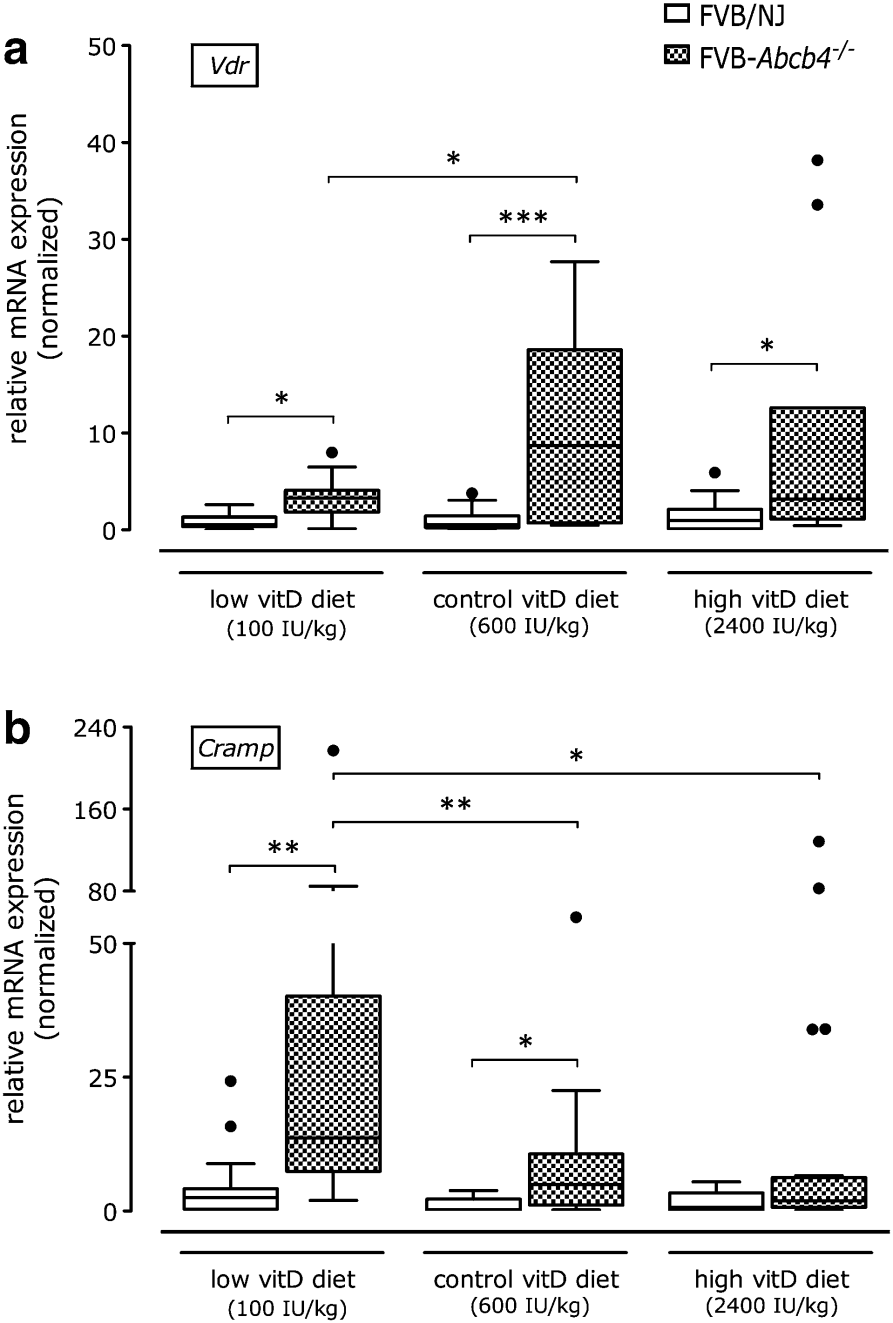
Relative hepatic mRNA expression levels of the (**a**) vitamin D receptor (*Vdr*) and (**b**) cathelicidin (*Cramp*) genes in *Abcb4* ^−*/*−^ and wild-type mice fed different vitamin D diets for 12 weeks. Data are normalized based on the expression of wild-type mice fed the vitamin D control diet (600 IU/kg food). *p < 0.05, **p < 0.01, ***p < 0.001

The hepatic gene expression of *Vdr* was increased in ABCB4-deficient mice compared with the corresponding wild-type controls in all diet groups. Similar to *Tgf*-*β1* and *Timp1* expression, vitamin D-deficient *Abcb4* ^−*/*−^ mice displayed lower expression levels of *Vdr* as compared to *Abcb4* ^−*/*−^ mice on the control diet (Fig. 6a). Panel B of Fig. 6 illustrates that hepatic gene expression of the antimicrobial peptide cathelicidin (*Cramp*) was increased in *Abcb4* ^−*/*−^ animals on control and low vitamin D diets, whereas knockout mice fed the high vitamin D diet did not differ from wild-type animals. The highest *Cramp* expression levels were observed in vitamin D-deficient *Abcb4* ^−*/*−^ mice.

As circulating vitamin D levels depend on the *Abcb4* genotype and diet (Fig. 1), we assessed expression differences of the major vitamin D hydroxylase *Cyp2r1* and *Cyp27a1* in liver. The mRNA levels of both enzymes were markedly increased in *Abcb4* ^−*/*−^ mice challenged with low and high vitamin D diets (data not shown). The expression of the genes *Cyp7a1* and *Cyp8b1*, encoding the rate-limiting enzymes for bile acid synthesis and conversion to cholic acid, respectively, tended to decrease in *Abcb4* ^−/−^ mice on diets with higher vitamin D contents, but no significant differences were observed (data not shown).

### Vitamin D supplementation does not appear to influence hepatic adherens junction integrity

**Fig. 7:**
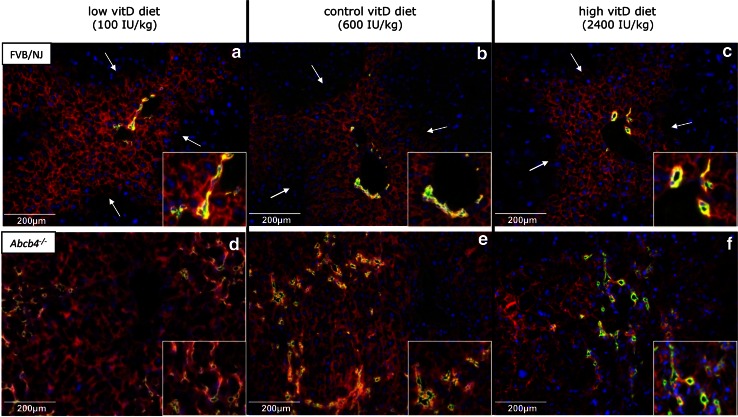
Double immunofluorescence staining for K19 (in green) and E-cadherin (in *red*) of livers from *Abcb4* ^−*/*−^ and wild-type mice fed different vitamin D diets for 12 weeks. At the hepatocyte level, predominant E-cadherin expression occurs in zone I of the liver lobe in wild-type animals (indicated by white arrows, panels **a**–**c**), whereas diffuse E-cadherin expression is present in *Abcb4* ^−*/*−^ mice (panels **d**–**f**). *bd* bile duct, *pv* portal vein. (Color figure online)

As determined by double immunofluorescence microscopy (Fig. 7), genotype-specific changes of bile duct integrity could be observed. *Abcb4* ^−*/*−^ animals from all vitamin D diet groups displayed an increase in ductular proliferation, as shown by the higher number of K19-positive cholangiocytes and overexpression of E-cadherin. At the hepatocyte level, a diffuse E-cadherin expression within the liver lobe was detected in *Abcb4* ^−*/*−^ mice (Fig. 7d–f), which contrasted with the findings in wild-type controls. However, an influence of dietary vitamin D intake on changes in the E-cadherin distribution pattern on the hepatocyte or even bile duct level was not apparent in either strain.

## Discussion

In this study, we examined the modulatory effects of dietary vitamin D supplementation on biliary fibrosis in vivo in *Abcb4* ^−*/*−^ mice. The following major results were demonstrated: (1) vitamin D deficiency is associated with increased collagen accumulation and fibrogenesis in this preclinical model; (2) a vitamin D-sufficient diet ameliorates chronic liver injury; and (3) high dose vitamin D supplementation exerts a further benefit only by reduction of aminotransferase activities. Taken together, these findings suggest that low vitamin D levels aggravate liver fibrosis, but supranormal vitamin D levels have no additive antiinflammatory or -fibrotic effects in *Abcb4* ^−*/*−^ mice. This may indicate that vitamin D deficiency should be avoided to decrease the risk of progressive fibrosis in chronic cholangitis and that an adequate vitamin D intake has, at least to some extent, antiinflammatory and anti-fibrotic effects. These observations are relevant for future translational studies, because chronic cholestatic liver injury in humans is frequently accompanied by vitamin D deficiency [7, 28]. More specifically, *Abcb4* ^−*/*−^ mice receiving the low vitamin D diet developed more advanced stages of fibrosis as compared to mice consuming diets with higher vitamin D contents. Of note, maximum vitamin D supplementation did not further improve the fibrosis scores in our model, but in comparison to the control group it ameliorated hepatocellular damage, as illustrated by lower serum aminotransferase activities. These observations are also reflected by enhanced hepatic collagen accumulation in *Abcb4* ^−*/*−^ mice with vitamin D deficiency, whereas the lowest collagen contents were observed in mice receiving the high vitamin D diet. The mRNA expression analyses did not identify differences in collagen expression, pointing to vitamin D-dependent post-transcriptional regulatory mechanisms. On the other hand, in vitro studies and a rat model of toxin-induced hepatic fibrosis have demonstrated possible antifibrotic effects of vitamin D supplementation through diminished hepatic stellate cell (myofibroblast) activation and collagen expression [29–31]. These different observations might be due to the diverse experimental setups, in particular homogeneous cell cultures versus heterogeneous cell populations from total liver, given that in cholestatic disorders portal fibroblasts are predominantly responsible for hepatic collagen deposition [32].

Despite identical dietary intake, *Abcb4* ^−*/*−^ mice demonstrated lower vitamin D serum concentrations on control chow diet as compared to wild-type mice. Serum vitamin D levels increased markedly in *Abcb4* ^−*/*−^ mice receiving the high vitamin D diet, whereas it remained constant in wild-type mice. These findings allude to a potential dysregulation of intestinal vitamin D uptake. Additionally, we observed higher expression of the vitamin D activating enzymes *Cyp2r1* and *Cyp27a1* in livers of ABCB4-deficient mice on the high vitamin D diet. Vitamin D levels are regulated through degradation via 24-hydroxylase, and vitamin D induces the expression of this enzyme, thus controlling its own catabolism [33, 34]. Of note, bile acids such as lithocholic acid have been shown to decrease this stimulatory effect of vitamin D and influence a broad spectrum of signaling pathways via the nuclear receptors VDR, FXR and PXR [35–38]. In fact, minor changes in bile salt composition have been shown to affect hepatic fibrogenesis in ABCB4-deficient mice [39], although the expression of the genes encoding the rate-limiting enzymes for bile salt synthesis (*Cyp7a1*) and conversion to the hydrophobic cholic acid (*Cyp8b1*) showed no major differences across the groups. Further studies, however, would be required to elucidate the molecular mechanisms underlying the genotype-diet interactions.

The expression levels of the fibrosis markers *Col1α2*, *Timp1* and *Tgf*-*β1* did not strictly correlate with the severity of liver injury and fibrosis. Some of these discrepancies could be related to the fact that we measured mRNA levels in total liver, although vitamin D might exert specific effects on different hepatic cell populations. Altered vitamin D-VDR signaling, which has been reported in cholestatic conditions, was corroborated in this study, as illustrated by increased *Vdr* expression in ABCB4-deficient as compared to wild-type mice. Furthermore, *Vdr* expression was reduced in *Abcb4* ^−*/*−^ mice on the low vitamin D diet, and these animals displayed the highest fibrosis scores. Recently, elegant mechanistic studies have demonstrated that the activation of VDR antagonizes TGF-β-induced recruitment of SMAD3 via co-occupation of regulatory sites in key profibrogenic genes (including *COL1A2*, *TIMP1* and *TGFB1*) in the presence of TGF-β [31]. In this context it is interesting to note that in comparison to *Abcb4* ^−*/*−^ mice on control diet, our data show lower *Tgf*-*β1* expression levels both in vitamin D deficiency and upon excess dietary supplementation, illustrating the interaction of vitamin D and TGF-β signaling in the setting of biliary inflammation and fibrosis.

Given the potential antiinflammatory effects of vitamin D, we also assessed the hepatic expression of the antimicrobial peptide cathelicidin (*Cramp*) [40]. Since the vitamin D response element is absent in the murine *Cramp* promoter (in contrast to humans) [41], we conclude that the decrease of *Cramp* expression upon vitamin D supplementation to levels observed in healthy controls is more likely to reflect the amelioration of hepatic inflammation than direct effects on *Cramp*. Further investigations into the regulation of antimicrobial peptides in sclerosing cholangitis would be interesting but were beyond the scope of this study.

To protect parenchymal liver cells from toxic bile compounds, epithelial cells form a physical barrier by apical junctional complexes (tight junction and adherens junction). Distinct functional and morphological alterations of these junctional complexes have been described in cholestasis in both mice and humans [19, 42]. Since vitamin D has been shown to be involved in the regulation of apical junctional complexes in other organs [43–45], Firrincieli et al. [46] recently investigated the role of VDR in the maintenance of bile duct integrity in mice with biliary-type injury. Their study demonstrated altered E-cadherin staining and loss of cell adhesion in biliary epithelial cells in *Vdr* ^−/−^ mice subjected to bile duct ligation [46]. Therefore, we also assessed hepatic adherens integrity in our model, which revealed genotype-specific differences in biliary epithelial cell proliferation and E-cadherin staining. However, vitamin D-dependent effects could not be substantiated. Nevertheless, this does not exclude the possibility that truncated E-cadherin, which was shown to be present in higher amounts in *Vdr* ^−*/*−^ mice [46], might be relevant.

In conclusion, our findings indicate that vitamin D modulates biliary injury and fibrogenesis in vivo and that the severity of biliary fibrosis in *Abcb4* ^−*/*−^ mice is influenced by vitamin D status. In this model, vitamin D deficiency appears to aggravate liver fibrosis; however, the beneficial effects of vitamin D supplementation are limited. We speculate that excess vitamin D supplementation does not fully protect against liver fibrosis, but an adequate vitamin D intake abates hepatic injury and confers antifibrotic effects in patients with cholestatic liver diseases.

## Electronic supplementary material

Below is the link to the electronic supplementary material.

Supplementary figure 1. Vitamin D metabolism (for details see Introduction). Abbreviations: CYP27A1, cytochrome P450, family 27, subfamily A, polypeptide 1; CYP27B1, cytochrome P450, family 27, subfamily B, polypeptide 1; CYP2R1, cytochrome P450, family 2, subfamily R, polypeptide 1; RXR, the retinoid X receptor; VBP, vitamin D-binding protein; VDR, vitamin D receptor; VDRE, vitamin D responsive elements

Supplementary Fig. 2. Serum calcium concentrations in *Abcb4* ^−*/*−^ and wild-type mice fed different vitamin D diets for 12 weeks.

Supplementary material 1 (DOC 561 kb)

Supplementary material 2 (DOC 136 kb)

Supplementary material 3 (DOC 540 kb)

Supplementary material 4 (TIFF 1521 kb)

Supplementary material 5 (TIFF 1521 kb)
