# Supplementary material for: Vitamin D modulates biliary fibrosis in ABCB4-deficient mice
Source: Hepatol Int. 2014 Jun 21;8(3):443–52. doi: 10.1007/s12072-014-9548-2 (PMC4148166; doi:10.1007/s12072-014-9548-2)
Supplement: Supplementary file 2 — Supplementary material 2 (DOC 136 kb) [file 12072_2014_9548_MOESM2_ESM.doc]

**Supplementary table 3:** Nutrient composition of the high vitamin D diet.

| **Nr.** | **Ingredients** | **Unit** |  | **Content** |  |
| --- | --- | --- | --- | --- | --- |
| 1 | Rohprotein / Crude Protein | mg/kg |  | 172750.675 |  |
| 2 | Rohfett / Crude Fat | mg/kg |  | 50854.935 |  |
| 3 | Rohfaser / Crude Fibre | mg/kg |  | 31008.805 |  |
| 4 | Rohasche / Crude Ash | mg/kg |  | 54903.335 |  |
| 5 | Feuchtigkeit / Moisture | mg/kg |  | 90098.500 |  |
| 7 | Disaccharide(s) | mg/kg |  | 98000.000 |  |
| 8 | Polysaccharide(s) | mg/kg |  | 483212.150 |  |
| 9 | Umsetzb. Energie/Metab. Energy | kcal/kg |  | 3485.013 |  |
| 10 | Lysin / Lysine | mg/kg |  | 17402.897 |  |
| 11 | Methionin / Methionine | mg/kg |  | 10689.294 |  |
| 12 | Cystin / Cystine | mg/kg |  | 3197.551 |  |
| 13 | Threonin / Threonine | mg/kg |  | 7156.615 |  |
| 14 | Tryptophan | mg/kg |  | 1977.374 |  |
| 15 | Arginin / Arginine | mg/kg |  | 9831.933 |  |
| 16 | Histidin / Histidine | mg/kg |  | 5277.640 |  |
| 17 | Isoleucin / Isoleucine | mg/kg |  | 7225.329 |  |
| 18 | Leucin / Leucine | mg/kg |  | 14770.647 |  |
| 19 | Phenylalanin / Phenylalanine | mg/kg |  | 7175.191 |  |
| 20 | Valin / Valine | mg/kg |  | 3299.219 |  |
| 21 | Alanin / Alanine | mg/kg |  | 2533.174 |  |
| 22 | Asparaginsäure / Aspartic acid | mg/kg |  | 3587.512 |  |
| 23 | Glutaminsäure / Glutamic acid | mg/kg |  | 23687.245 |  |
| 24 | Glycin / Glycine | mg/kg |  | 3138.587 |  |
| 25 | Prolin / Proline | mg/kg |  | 12769.008 |  |
| 26 | Serin / Serine | mg/kg |  | 5271.163 |  |
| 27 | Tyrosin / Tyrosine | mg/kg |  | 9287.817 |  |
| 28 | Vitamin A | I.E./kg |  | 15000.000 |  |
| 29 | **Vitamin D3** | I.E./kg |  | 2400.000 |  |
| 30 | Vitamin E | mg/kg |  | 180.400 |  |
| 31 | Vitamin K3 als/as Menadion(e) | mg/kg |  | 10.000 |  |
| 32 | Vitamin B1 | mg/kg |  | 20.040 |  |
| 33 | Vitamin B2 | mg/kg |  | 20.322 |  |
| 34 | Vitamin B6 | mg/kg |  | 15.034 |  |
| 35 | Vitamin B12 | mg/kg |  | 0.041 |  |
| 36 | Nikotinsäure / Nicotinic acid | mg/kg |  | 50.170 |  |
| 37 | Pantothensre./Pantothenic acid | mg/kg |  | 50.106 |  |
| 38 | Folsäure / Folic acid | mg/kg |  | 10.01200 |  |
| 39 | Biotin | mg/kg |  | 0.201 |  |
| 40 | Cholinchlorid/Choline chloride | mg/kg |  | 1011.500 |  |
| 42 | P-Aminobenzoesre./Benzoic acid | mg/kg |  | 100.000 |  |
| 43 | Inosit / Inositol | mg/kg |  | 111.000 |  |
| 44 | Vitamin C | mg/kg |  | 21.000 |  |
| 45 | Calcium | mg/kg |  | 9539.831 |  |
| 46 | Ges.Phosphor / Phosphorus | mg/kg |  | 7533.017 |  |
| 47 | Verd.Phosphor/Digest.Phosporus | mg/kg |  | 7208.524 |  |
| 48 | Magnesium | mg/kg |  | 666.855 |  |
| 49 | Natrium / Sodium | mg/kg |  | 2501.037 |  |
| 50 | Kalium / Potassium | mg/kg |  | 7171.960 |  |
| 51 | Schwefel / Sulfur | mg/kg |  | 2793.480 |  |
| 52 | Chlor / Chlorine | mg/kg |  | 3630.000 |  |

| **Nr.** | **Ingredients** | **Unit** |  | **Content** |  |
| --- | --- | --- | --- | --- | --- |
| 53 | Eisen / Iron | mg/kg |  | 178.602 |  |
| 54 | Mangan / Manganese | mg/kg |  | 100.891 |  |
| 55 | Zink / Zinc | mg/kg |  | 29.304 |  |
| 56 | Kupfer / Copper | mg/kg |  | 5.645 |  |
| 57 | Jod / Iodine | mg/kg |  | 0.450 |  |
| 58 | Molybdän / Molybdenum | mg/kg |  | 0.198 |  |
| 59 | Fluor / Fluorine | mg/kg |  | 4.170 |  |
| 60 | Selen / Selenium | mg/kg |  | 0.334 |  |
| 61 | Kobalt / Cobalt | mg/kg |  | 0.148 |  |
| 63 | Caprinsäure C-10:0 | mg/kg |  | 2.500 |  |
| 64 | Laurinsäure C-12:0 | mg/kg |  | 2.500 |  |
| 65 | Myristinsäue C-14:0 | mg/kg |  | 2.500 |  |
| 66 | Pentadecansäure C-15:0 | mg/kg |  | 2.500 |  |
| 67 | Palmitinsäure C-16:0 | mg/kg |  | 2700.000 |  |
| 68 | Palmitoleinsäure C-16:1 | mg/kg |  | 2.500 |  |
| 69 | Margarinsäure | mg/kg |  | 2.500 |  |
| 70 | Stearinsäure C-18:0 | mg/kg |  | 1250.000 |  |
| 71 | Ölsäure C-18:1 | mg/kg |  | 10950.000 |  |
| 72 | Linolsäure C-18:2 | mg/kg |  | 35050.000 |  |
| 73 | Linolensäure C-18:3 | mg/kg |  | 150.000 |  |
| 74 | Arachinsäure C-20:0 | mg/kg |  | 250.000 |  |
| 75 | Eicosaensäure C-20:1 | mg/kg |  | 250.000 |  |
| 76 | Eicosadiensäure C-20:2 | mg/kg |  | 250.000 |  |
| 77 | Arachidonsäure C-20:4 | mg/kg |  | 2.500 |  |
| 78 | Eicosapentaensäure C-20:5 | mg/kg |  | 2.500 |  |
| 79 | Behensäure C-22:0 | mg/kg |  | 250.000 |  |
| 81 | Docosahexaensäure C-22:6 | mg/kg |  | 2.500 |  |
| 82 | Tricosansäure | mg/kg |  | 2.500 |  |
| 83 | Nervonsäure C-24:1 | mg/kg |  | 2.500 |  |
| 87 | Erucasäure C-22:1 | mg/kg |  | 2.500 |  |
| 111 | Aluminium | mg/kg |  | 3.732 |  |
| 155 | Volumen / Volume | kg |  | 1000.000 |  |

Altromin Spezialfutter GmbH & Co. KG Im Seelenkamp 20 -D-32791 Lage Tel.: +49 (0)5232/6088-0 -Fax: +49 (0)5232/6088-20 E-Mail: info@altromin.de -http://www.altromin.de
