# Supplementary material for: Vitamin D modulates biliary fibrosis in ABCB4-deficient mice
Source: Hepatol Int. 2014 Jun 21;8(3):443–52. doi: 10.1007/s12072-014-9548-2 (PMC4148166; doi:10.1007/s12072-014-9548-2)
Supplement: Supplementary file 3 — Supplementary material 3 (DOC 540 kb) [file 12072_2014_9548_MOESM3_ESM.doc]

Supplementary table 2: Nutrient composition of the control vitamin D diet.
