# Supplementary figures and images for: Vitamin D modulates biliary fibrosis in ABCB4-deficient mice
Source: Hepatol Int. 2014 Jun 21;8(3):443–52. doi: 10.1007/s12072-014-9548-2 (PMC4148166; doi:10.1007/s12072-014-9548-2)

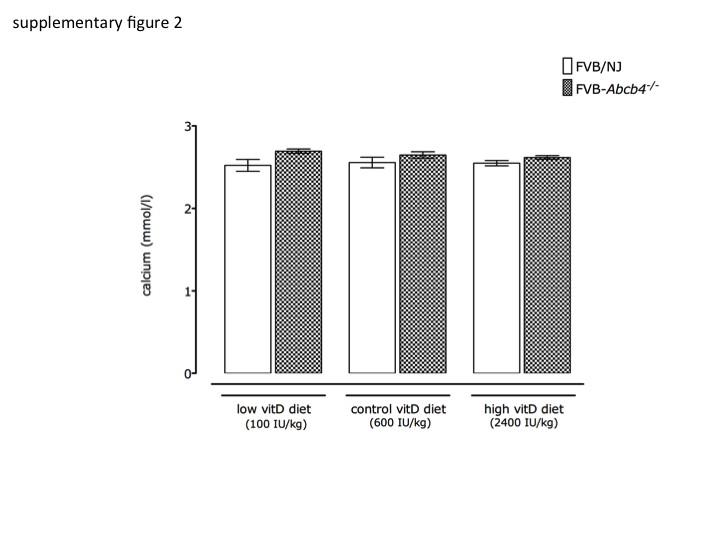

Supplement: Supplementary file 4 — Supplementary material 4 (TIFF 1521 kb) [file 12072_2014_9548_MOESM4_ESM.tiff]

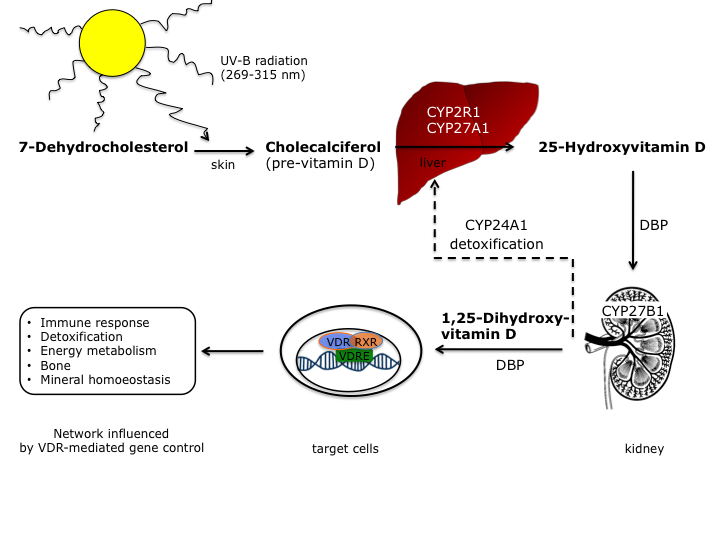

Supplement: Supplementary file 5 — Supplementary material 5 (TIFF 1521 kb) [file 12072_2014_9548_MOESM5_ESM.tiff]
